# Whole-genome resequencing of large yellow croaker (*Larimichthys crocea*) reveals the population structure and signatures of environmental adaptation

**DOI:** 10.1038/s41598-021-90645-1

**Published:** 2021-05-27

**Authors:** Tetsuo Kon, Liyi Pei, Ryota Ichikawa, Chunyan Chen, Ping Wang, Ikuyo Takemura, Yingying Ye, Xiaojun Yan, Baoying Guo, Weiye Li, Hagai Nsobi Lauden, Hiromasa Tabata, Hao Pan, Yoshihiro Omori, Atsushi Ogura, Lihua Jiang

**Affiliations:** 1 National Engineering Research Center of Marine Facilities Aquaculture, Zhejiang Ocean University, Zhoushan, 316022 China; 2 Genomic Diversity Laboratory, Graduate School of Bioscience, Nagahama Institute of Bioscience and Technology, Nagahama, Shiga 526-0829 Japan; 3 Laboratory of Functional Genomics, Graduate School of Bioscience, Nagahama Institute of Bioscience and Technology, Nagahama, Shiga 526-0829 Japan; 4 Fisheries Research Institute of Zhoushan, Zhoushan, China

**Keywords:** Biochemistry, Ecology, Evolution, Genetics

## Abstract

Large yellow croaker is an economically important fish in China and East Asia. Despite its economic importance, genome-wide adaptions of domesticated large yellow croaker are largely unknown. Here, we performed whole-genome resequencing of 198 individuals of large yellow croaker obtained in the sea or from farmers in Zhoushan or Ningde. Population genomics analyses revealed the genetic population structure of our samples, reflecting the living environment. Each effective population size is estimated to be declining over generations. Moreover, we identified genetically differentiated genomic regions between the sea-captured population in the Zhoushan Sea area and that of the Ningde Sea area or between the sea-captured population and the farmed population in either area. Gene ontology analyses revealed the gene groups under selective sweep for the adaptation to the domesticated environment. All these results suggest that individuals of the large yellow croaker populations show genomic signatures of adaptation to different living environments.

## Introduction

*Larimichthys crocea*, more commonly known as the large yellow croaker, is a sciaenid fish species. The ecological and genetic studies of large yellow croakers are important because they are economically important in Chinese coastal regions. Wild large yellow croaker was originally distributed from the southern Yellow Sea to the South China Sea^1^. However, wild stocks of the large yellow croaker have suffered severely from overfishing and are on the brink of extinction. Large yellow croaker was initially domesticated in the early 1980s. The annual yield of large yellow croaker aquaculture in China has been greater than that of any other domesticated marine fish^2^. After the initial artificial breeding attempts were successful in the 1980s, enhancement and release were also carried out. It has been reported that the first enhancement and release of 16,200 individuals took place in the Ningde Sea as early as 1987. This was followed by a rapid increase with the release of millions of large yellow croaker carried out annually^3^. It is hard to define whether a large yellow croaker captured in the sea is wild or domesticated based on the above background. Therefore, in this study, we defined the populations of our samples as sea-captured population and the farmed population.

The culture performance of farmed large yellow croaker populations has declined, mainly because of irrational artificial breeding, inbreeding, and blind introduction. They have caused the degradation of genetic resources and hybrid germplasm in the large yellow croaker^4^. Previous genetic studies of the population structure of large yellow croaker are available for both domesticated and sea-captured populations; however, these studies have been limited by putative neutral markers; e.g., microsatellites^5^ and single nucleotide polymorphism (SNP) loci^6^ including narrow regions of the genome^7^. This is not enough genetic data to describe the structure of the population. In this study, whole-genome resequencing of the 198 croakers were performed to obtain a better information of the critical uncertainties associated with population structure, genetic diversity and the analysis of mixed stocks across the domesticated and sea-captured populations of large yellow croaker.

As mentioned above, for each generation over the last decades, large yellow croaker has been exposed to directional selection for an increasing number of economically important traits, such as growth, anti-freeze capacity, desirable flesh characteristics and disease resistance^8,9^. Genetic improvement of strains of large yellow croaker for commercial aquaculture is in the process of establishment in China at the current time^10^. As a result, currently, there are differences in phenotypic traits between domesticated and sea-captured large yellow croaker^9^. These traits may render large yellow croaker a good model for investigating the variation response to different living environments. Recently, basic genome-wide scale studies on genetic improvement in the production traits of large yellow croaker were reported. Until 2019, genomic papers on the large yellow had been published: the draft genome of the large yellow croaker was published in 2014, indicating a well-developed innate immune system^1^. Ao et al combined genomic, transcriptomic and metabolomic data to analyse the multiple protective mechanisms involved in antioxidant functions, oxygen transport, immune defence, and osmotic and ionic regulation. The results mainly reveal molecular responses to hypoxia and air exposure in this croaker^2^. Moreover, a genome-wide association study of growth and body shape-related traits in large yellow croaker using ddRAD sequencing has also been reported^9^.

However, the molecular response of large yellow croaker to different living environments, i.e. sea-captured and farmed croakers, is still poorly understood. Similar to other vertebrates, the essential factor for domestication is the consistent control of reproduction every year in successive generations of fish, which are maintained and bred in captivity^11^. Therefore, in the domesticated environments, the fish life cycle must be completely closed in captivity, completely independent of wild sources including eggs, larvae^12^. However, as the sea living environment displays a limited production capacity^13^, food could be limited in the natural environment and the captured large yellow croaker have had to expend more energy catching prey in the sea environment. On the other hand, the farmed large yellow croaker is well-maintained using soybean feed given by breeders. Since there are genetic differences between farmed and sea-captured large yellow croaker, a wide range of phenotypic traits has been observed between them^14^. For example, the body of sea-captured croaker is slenderer than the farmed fish^9^. The body colour and flavour are also different between them^9^.

To understand the population structure of sea-captured and farmed large yellow croaker, as well as the molecular variation for adapting to different living environments that include natural sea environment and domesticated environment, we re-sequenced the genome of 198 croakers. The genetic structure was obtained and GO analysis was conducted. The results clearly showed the genetic structure and genes associated with the response to adaption to the domesticated environment. Our results provide a better understanding of the molecular and genetic basis of large yellow croaker and fish adaptation to the living environments.

## Results

### Whole genome resequencing of large yellow croaker populations

**Figure 1:**
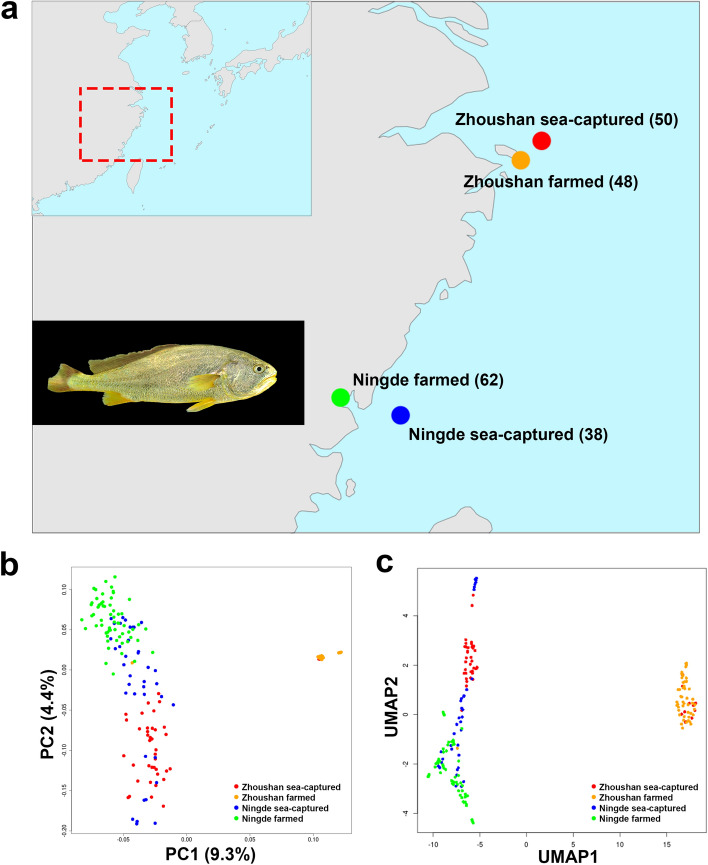
Population structure and relationship of large yellow croaker. (**a**) Geographic map indicating the sample origins of the large yellow croaker in this study. The gross appearance of a large yellow croaker individual is shown in the picture. The sampling area is highlighted by the red broken line. The dots of different color stand for different population. The number of individuals is given in parentheses after the population name. The geographical maps were generated by using R packages of maps v3.3.0 (https://cran.r-project.org/web/packages/maps) and mapdata v2.3.0 (https://cran.r-project.org/web/packages/mapdata). (**b**) PCA plot (PC1 and PC2) showing the genetic structure of the 198 large yellow croaker individuals. The degrees of explained variance is given in parentheses. Colors reflect the geographic regions in (**a**). (**c**) UMAP of the 198 large yellow croaker individuals. Colors reflect the geographic regions in (**a**).

We collected a total of 198 large yellow croaker individuals (Table S1). Of these, 50 individuals were captured in the Zhoushan Sea (the red dot in Fig. 1a) and 48 individuals had been farmed in Zhoushan (the orange dot in Fig. 1a). A further 38 individuals were captured in the Ningde Sea (the blue dot in Fig. 1a). and 62 individuals had been farmed in Ningde (the green dot in Fig. 1a). We performed whole-genome resequencing of these 198 large yellow croaker individuals. We obtained 1.42 Penta base-pairs of genomic DNA, representing about 11 × sequencing depth of the genome per individual. Raw reads were trimmed and aligned to the genome sequence. After variant calling and filtering, a total of 6,302,244 single nucleotide polymorphisms (SNPs) were identified. Using this SNP information, we performed the following population genomic analyses.

### Genetic population structure of the large yellow croaker individuals

In order to examine the genetic population structure of the large yellow croaker individuals, we performed principal component analysis (PCA). In the first component of the PCA, the Zhoushan farmed population separated from the other three populations (Fig. 1b). In the second component of the PCA, the Zhoushan sea-captured population formed a cluster. Also, the Ningde farmed population formed a cluster. The Ningde sea-captured population had a wider distribution than the other populations. Then, we performed uniform manifold approximation and projection (UMAP), a non-linear dimensionality method (Fig. 1c). The result of UMAP is similar to the result of PCA. UMAP showed that the Zhoushan farmed population formed a distinct cluster, and the Zhoushan sea-captured population and Ningde farmed population formed more scattered clusters. UMAP also showed that the Ningde sea-captured population had a wider distribution than the other populations.

**Figure 2:**
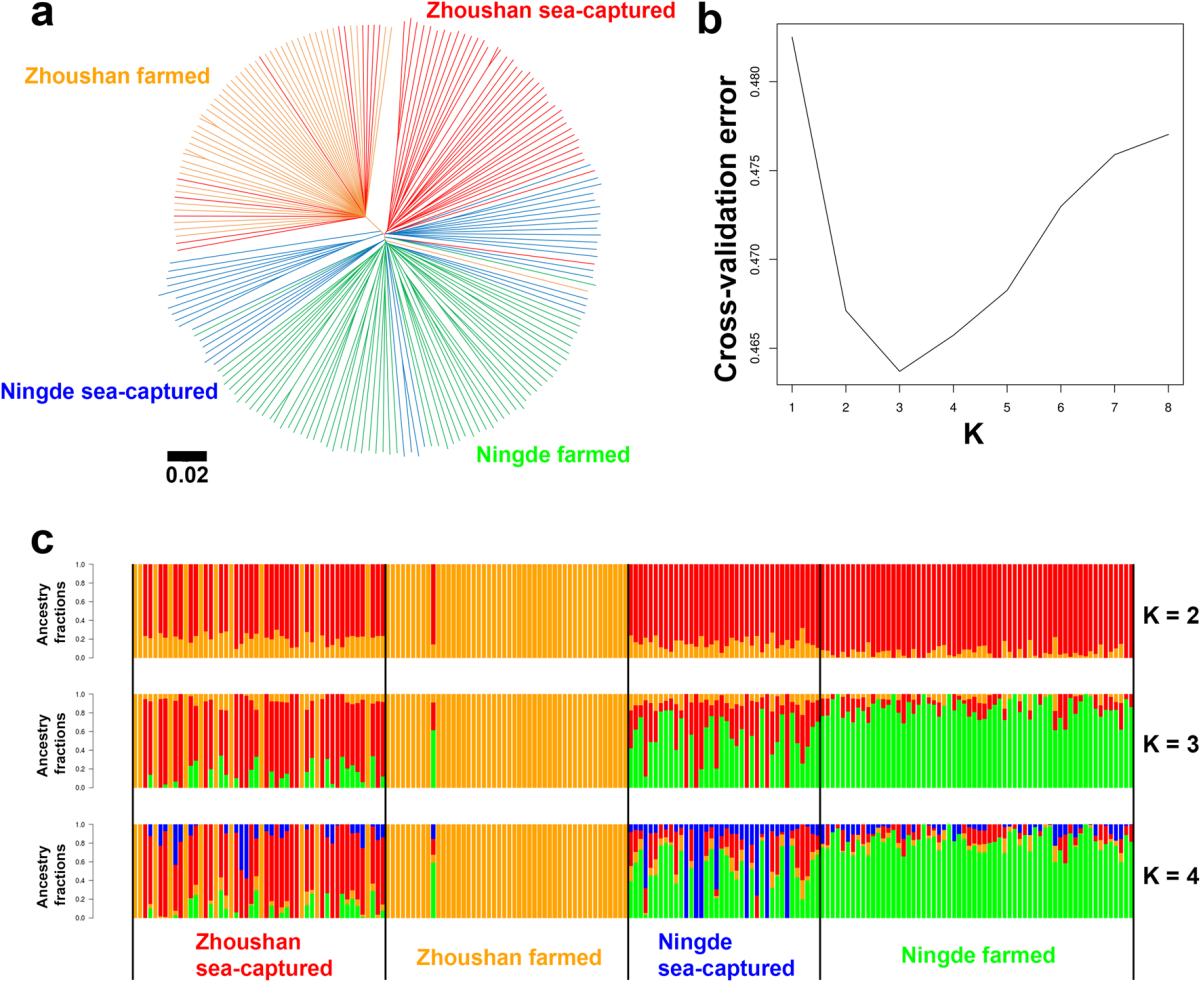
Neighbor-joining tree and admixture analysis using whole-genome SNP data. (**a**) Neighbor-joining tree of the 198 large yellow croaker individuals. The color scheme follows Fig. 1. The scale bar represents pairwise distances between different individuals. (**b**) Cross-validation error in the admixture analysis. The x-axis represents K values and the y-axis represents the corresponding cross-validation error. The cross-validation error was lowest at K = 3. (**c**) Admixture plot (K = 2, 3, 4) for the 198 large yellow croaker individuals. Each individual is shown as vertical bar divided into K colors. The color scheme follows Fig. 1. Individuals are arranged by population**.**

The evolutionary history of the individuals was inferred with the neighbour-joining (NJ) tree. The NJ tree contains two large groups (Fig. 2a). The first group was formed by the individuals of the Zhoushan farmed population plus several individuals of the Zhoushan sea-captured population. The other group was formed by the individuals in the other three groups. In this group, individuals of the Zhoushan sea-captured formed a distinct cluster from the individuals of the Ningde sea-captured population and those of the Ningde farmed population. The individuals of the Ningde sea-captured population and those of the Ningde farmed population together formed several small groups.

We performed unsupervised clustering analysis with ADMIXTURE to evaluate the relatedness of the populations. Cross-validation error was lowest at K = 3, suggesting that the population genetic structure of our samples is best modelled by considering the admixture of the three genetic components (Fig. 2b). The individuals of the Zhoushan farmed population are composed of relatively uniform genetic components (Fig. 2c). The individuals of the Ningde farmed population are composed of genetic components that are also relatively uniform but different from those of the Zhoushan farmed population. Both the individuals of the Zhoushan sea-captured population and those of the Ningde sea-captured population were a mixture of the three genetic components.

### Trends of effective population size

**Figure 3:**
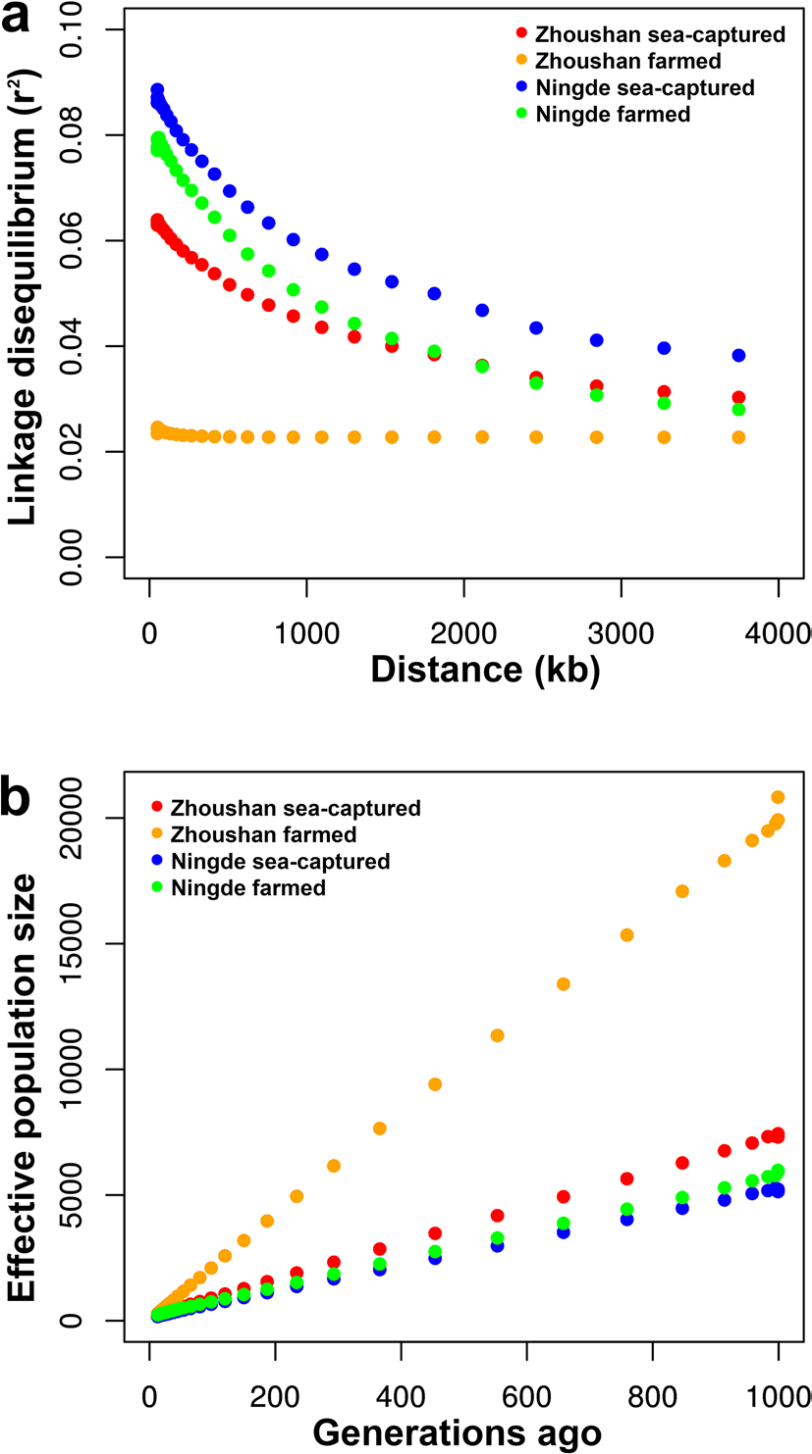
Trends of effective population sizes. (**a**) LD decay (r^2^) from 0 to 4000 kb for four populations. The x-axis represents marker distances between pairwise SNPs. The y-axis represents r^2^ values of linkage disequilibrium. (**b**) Effective population sizes of four populations over the past 1000 generations. All of the four populations showed decreasing trends.

We evaluated the extents of linkage disequilibrium for SNP pairs. The average r^2^ values of linkage disequilibrium decreased by increasing the marker distance between pairwise SNPs, with a rapidly declining trend observed over the first 500 kb (Fig. 3a). Using this information, we estimated the change of the effective population size over the past 1000 generations (Fig. 3b). All the four populations showed decreasing trends of effective population sizes, suggesting that their genetic diversities remain at a low level.

### Detection of putative genes associated with the adaptation to different sea environments of the Zhoushan Sea and Ningde Sea

**Figure 4:**
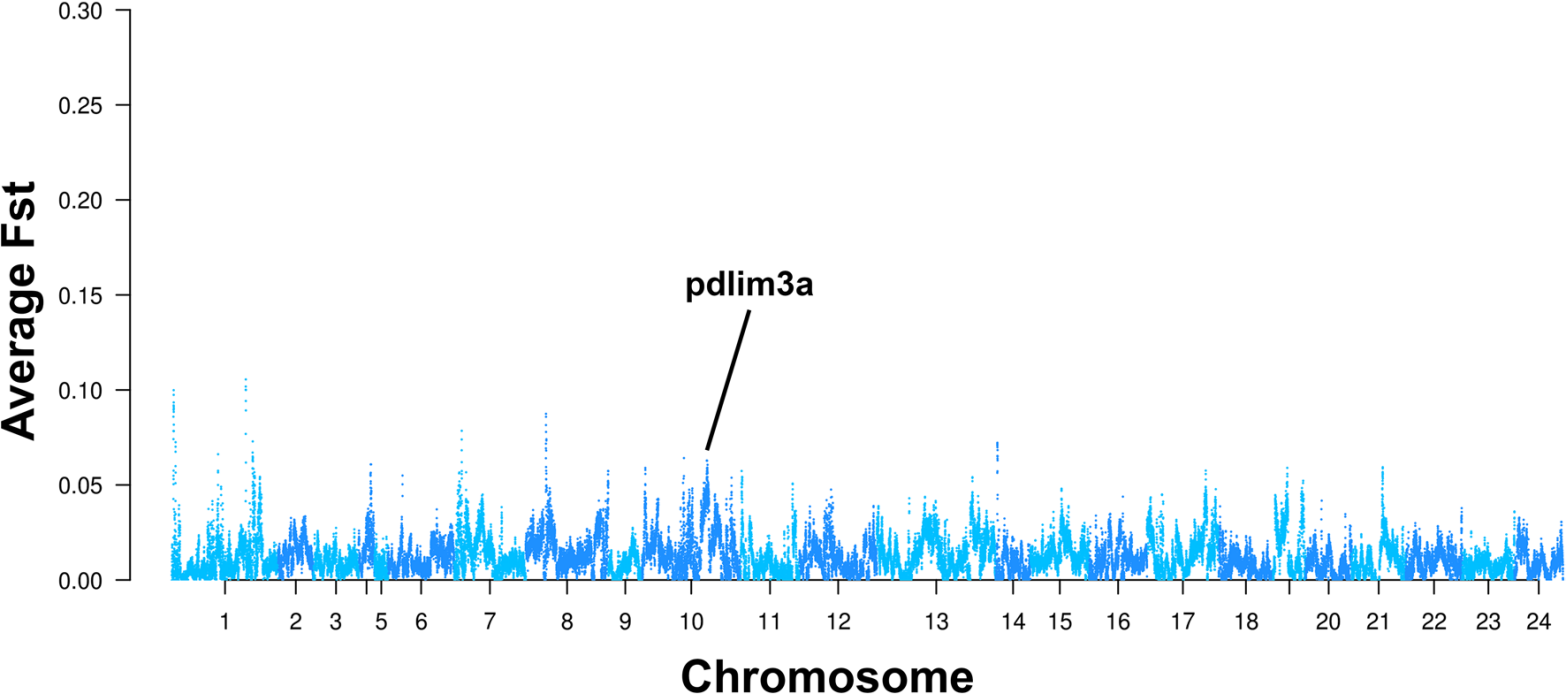
Genomic regions associated with regional differentiation of large yellow croaker between Zhoushan sea and Ningde sea. Manhattan plot for average Fst values in 40 kb windows with 10 kb steps between Zhoushan sea-captured population and Ningde sea-captured population. The x-axis represents chromosomal positions and the y-axis represents the average Fst values.

To identify the genetic markers to differentiate individuals of the Zhoushan sea-captured and Ningde sea-captured, we calculated fixation index (Fst) values for each SNP. We identified total 819 SNPs as genetic markers (Table S2). To identify the genes associated with adaptation to the different living environments between these two regions, we calculated average Fst values in 40 kb windows with 10 kb steps (Fig. 4). We identified 47 regions with significant Fst values. The total size of these regions is 3.6 Mb. The sizes of the significant regions were between 40 kb to 0.31 Mb. These regions contained 88 genes (Table S3). We categorised the functions of these genes based on their gene ontology (GO) term annotations (Table S4). These genes include those involved in muscle structure development (GO:0061061) such as pdlim3a (pdz and lim domain 3). This gene is located in the region from 26,673,301 to 26,662,947 bp on chromosome 10, and is reported to be highly expressed in muscle and involved in the crosslinking of actin filaments^15^. We identified three upstream variants of this gene which are located at 26,675,034 bp, 26,675,134 bp, and 26,678,221 bp on chromosome 10 (Fig. 4). We also identified one downstream variant located at 26,660,973 bp on chromosome 10. Besides muscle structure development (GO:0061061), there are also some enriched GO terms such as regulation of response to external stimulus (GO:0032101) and cell–cell signalling (GO:0007267).

### Detection of putative genes under selective sweep between the Zhoushan sea-captured population and farmed population

**Figure 5:**
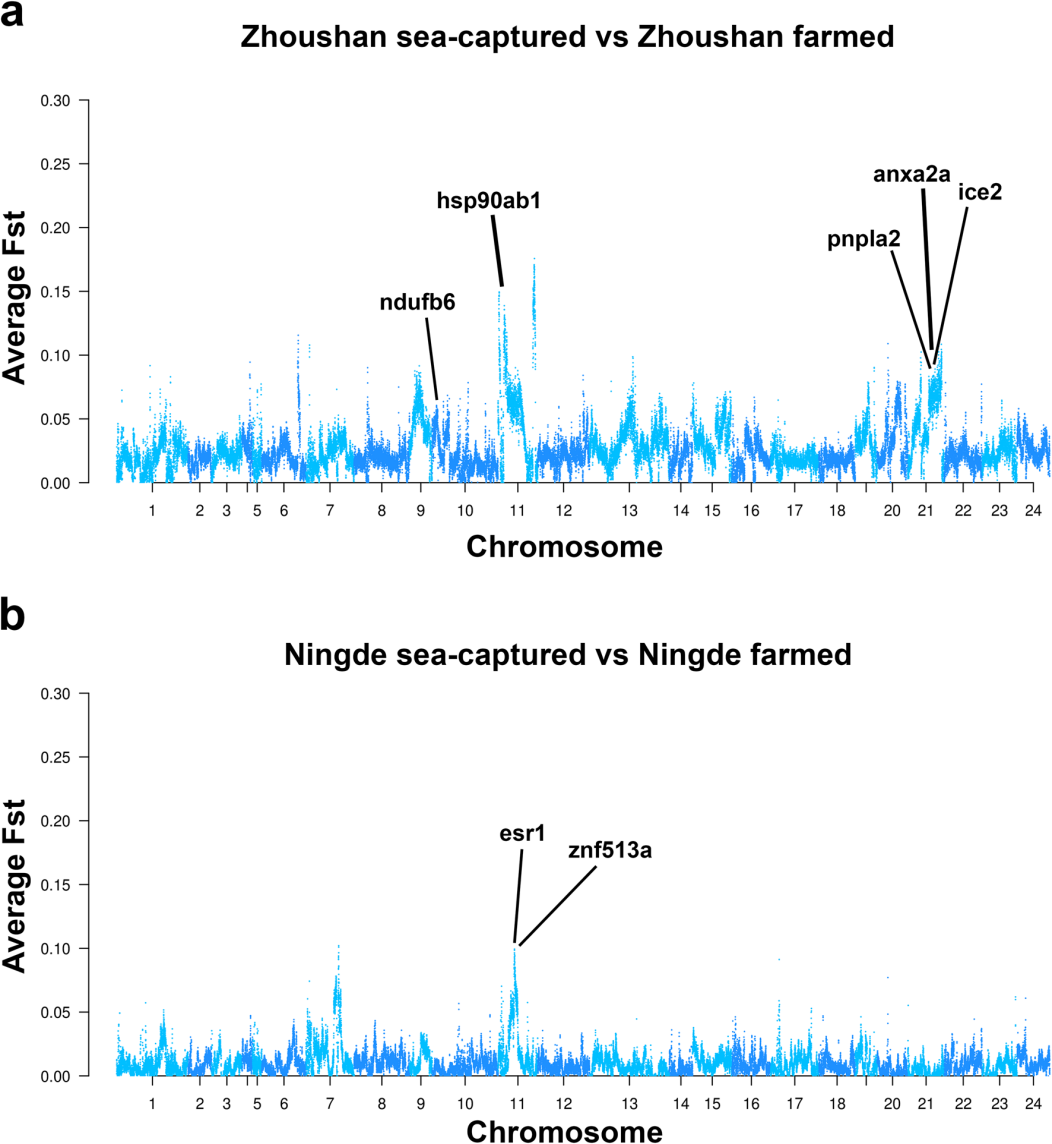
Genomic regions associated with domestication of large yellow croaker between Zhoushan sea or Ningde sea. (**a**) Manhattan plot for average Fst values in 40 kb windows with 10 kb steps between Zhoushan sea-captured and Zhoushan farmed. (**b**) Manhattan plot for average Fst values in 40 kb windows with 10 kb steps between Ningde sea-captured and Ningde farmed. The x-axis represents chromosomal positions and the y-axis represents the average Fst values.

To identify the genes under selective sweep in the domestication process, we analysed single Fst values for single SNPs and average Fst values in 40 kb windows with 10 kb steps separately both in the Zhoushan and Ningde regions. Between the Zhoushan sea-captured population and farmed population, we identified 23,862 SNPs with significant Fst values by single SNP analysis (Table S5). In the sliding window analysis, the number of significant regions was 317, and the total size of significant regions was 59 Mb (Fig. 5a). The sizes of significant regions were between 40 kb to 8.1 Mb. These regions contain 1709 genes (Table S6). We identified the strong peak of Fst signal on chromosome 11, which contains 423 genes such as hsp90ab1 (heat shock protein 90 alpha family class B member 1). GO analysis showed that genes involved in the regulation of fatty acid oxidation (GO:0031998), the steroid hormone mediated signalling pathway (GO:0043401), fatty acid metabolic processes (GO:0006631), membrane lipid metabolic processes (GO:0006643), regulation of fatty acid metabolic processes (GO:0019217), and long-chain fatty acid transport (GO:0015909). These GO terms include plenty of lipid metabolism-related genes such as ppara (peroxisome proliferator activated receptor alpha), pnpla2 (Patatin like phospholipase domain containing 2). It is worth mentioning that there were plenty of genes related to carbohydrate derivative metabolic processes (GO:1901135) with differences between the Zhoushan sea-captured population and farmed populations (Table S7). Additionally, a number of the growth relative genes include the developmental growth involved in morphogenesis (GO:0060560). Genes were found related to embryo development ending in birth or egg hatching (GO:0009792). Additionally, 47 genes related immune system development (GO:0002520) were obtained, such as taf3 (tata-box binding protein associated factor 3), irf7 (interferon regulatory factor 7) and rps7 (ribosomal protein s7) (Table S7).

Moreover, we found that anxa2a (annexin a2a; from 16,718,332 bp to 16,713,531 bp on chromosome 21) have a splice donor site variant at 16,715,408 bp on chromosome 21. This mutation is located at the fifth intron of anxa2a, and is predicted to lead to a premature truncation. The anxa2a gene encodes a phospholipid-binding protein, and is involved in variety of intracellular processes including endocytosis, exocytosis, membrane domain organisation, actin remodelling, signal transduction, protein assembly^16^. This batch of samples came from breeding selection for a freeze-resistant population. We identified nine downstream mutations (16,713,395 bp, 16,713,442 bp, 16,713,443 bp, 16,713,593 bp, 16,715,408 bp, 16,715,741 bp, 16,716,027 bp, 16,716,216 bp and 16,717,363 bp on chromosome 21) of ice2 (interactor of little elongation complex ELL subunit 2) gene, which is located in the region from 16,727,361 to 16,718,192 bp on chromosome 21. This gene is involved in cold acclimation and determines freezing tolerance^17^.

### Detection of putative genes under selective sweep between the Ningde sea-captured and farmed population

For the Ningde farmed population, we identified 660 SNPs with significant Fst values (Table S8). In the sliding window analysis, the number of significant regions was 42, and the total size of significant regions was 7.8 Mb (Fig. 5b). The sizes of significant regions were between 40 kb to 2.0 Mb. These regions contain 238 genes (Table S9). GO analysis showed identified genes related to the reproduction process such as female gonad development (GO:0008585), i.e. esr1 (estrogen receptor 1), foxo3 (forkhead box O3); the development of primary female sexual characteristics (GO:0046545) and embryonic appendage morphogenesis (GO:0035113), such as mbnl1 (muscle blind like splicing regulator 1); as well as embryonic limb morphogenesis (GO:0030326) and the response to steroid hormones (GO:0048545). Additionally, genes related to digestive tract development (GO:0048565) were enriched, such as hnf1b (hnf1 homeobox b) (Table S10)﻿. As per the results of SNPs with the highest Fst analysis between the Ningde sea-captured and farmed population, we identified a downstream variant of esr1, which is located at 9,103,629 bp on chromosome 11. This gene is located in the region from 9,129,853 and 9,108,464 bp on chromosome 11 and encodes estrogen receptor 1, which plays a critical role in responding to steroid hormones (Fig. 5b). Genes involved in visual system development (GO:0150063) such as prox1 (prospero-related homeobox1), nr2e1 (nuclear receptor subfamily 2 group e member 1) and znf513a (zinc finger protein 513a) were also enriched. The znf513a gene is located in the region from 11,664,515 to 11,657,703 bp on chromosome 11 and has a downstream variant located at 11,652,743 bp on this chromosome (Fig. 5b).

## Discussion

As the large yellow croaker wild population collapsed in the 1970s, artificial breeding was established in China in the 1980s as an important fish resource in China, with millions of enhanced releases conducted annually^18^. There are plenty of studies that have investigated productive characters such as growth and immunity^19^. On the other hand, studies have indicated that the genetic structure of large yellow croaker reared in captivity is different from that of captured populations and further expresses concerns over the genetic diversity of local populations perturbed by large-scale hatchery releases^20^; however, these studies were limited to microsatellite markers and SNP loci, which did not provide an in-depth discussion on the impact of domesticated programs on the large yellow croaker genome. Not much is understood about the impact of decades of domesticated on the genomes and phenomes of large yellow croaker. Further studies on whole genome data may provide deeper insight into the current status of large yellow croaker genomes and its relation to phenotypic traits.

The current study analysed the large yellow croaker genome based on whole genome population data. For this, 198 large yellow croaker genomes were collected from four different sea-captured and farmed populations. The structures of the four different large yellow croaker populations were investigated by PCA, admixture and phylogenetic tree analysis. Population stratification was clearly observed in all analyses, with decreased genetic diversity in the domesticated populations (Figs. 1 and 2), which is a possible outcome of a breeding program for selecting individuals. After observing that the four isolated populations were indeed distinguishable to some extent, a selective sweep analysis was performed. Here, it is important to note that the sea-captured populations were collected from the Zhoushan Sea and the Ningde Sea area, while the Zhoushan farmed population was collected from an anti-freeze selection breeding pool. Based on the PCA and structure analysis, the results clearly divided into four groups even though the diversity was low. This may be due to high degree of artificial anti-freeze selection in the large yellow croaker Zhoushan farmed population, which is not surprising with this stock, given that there is more selection by artificial breeding than the Ningde farmed population. At the same time, we analysed the Fst based on the SNPs located in the selective sweep region that had a significantly longer extension of the haplotype the nucleotide diversity and highly enriched GO terms were identified.

The results that Zhoushan sea-captured is close to Ningde farmed population on the PCA plot and the UMAP plot are supported by the history of the releasing of large yellow croaker fry from Ningde farmed population to the Zhoushan sea area (Fig. 1b,c). On the other hand, Zhoushan sea-captured population has a different genetic component from Ningde sea-captured population and Ningde farmed population in the NJ tree (Fig. 2a) and the admixture analysis (Fig. 2c). Zhoushan sea-captured population has genetic components colored in red and orange in Fig. 2c. Ningde sea-captured population and Ningde farmed population have more genetic component colored in green in Fig. 2c. This partial difference between PCA/UMAP and NJ tree/admixture results is probably due to the difference in their analytical sources. PCA and UMAP based on the top five principal components consider a part of the total variance of the data while NJ tree and admixture analysis summarize the entire data. Taken together, although the effects of the release of fry from Ningde farmed population to Zhoushan sea cannot be completely ruled out, the individuals from Zhoushan sea-captured population seems to still have a distinct genetic background from the Ningde farmed population.

The effective population size was analysed, and we estimated the history of the effective population size over the past 1000 generations (Fig. 3b). All four populations showed decreasing trends of the effective population size, suggesting that their genetic diversities remain at a low level. This indicates that they have all experienced a strong reduction in population size. Such a reduction in size creates extensive genetic drift, which alone can explain the genetic differentiation of these populations.

Another highlight of this study is the selection of gene mutations in domesticated croaker. Some interesting variation signatures were found in the farmed population. For the Ningde farmed stock, the first breeding of large yellow croaker was established in Ningde in the 1980s, and the first artificial release was carried out in the Ningde Sea^18^. This suggests that this area has the longest domesticated and largest-scale release history; at the same time, soybean meal was used as the feed in the large yellow croaker industry^21^. This is why there are plenty of variations in genes, such as carbohydrate derivative metabolic processes and fatty acid metabolic processes, that are highly associated with energy metabolism between the Zhoushan and Ningde farmed populations. Additionally, digestive tract development genes were different between the captured and domesticated were also obtained, which is also easily explained.

Apart from metabolic pathways, we also discovered GO terms related to developmental processes in the two farmed populations. SNPs in genes associated with female gonad development, gonad developmental growth involved in morphogenesis, embryonic limb morphogenesis and the response to steroid hormones were frequently observed. This may be evidence of artificial selection affecting the developmental processes or physical characteristics of farmed large yellow croaker, which grows faster and tends to mature earlier than the sea-captured population.

Moreover, differences were observed in genes involved in the sensory system such as visual related genes. Most large yellow croaker are raised in intensive cages indoors, such that the high cage culture density results in a stressed living environment. For example, the light is weaker than in the sea environment, which may explain some variations in genes. Of note, anxa2a has a splice donor mutation in the Zhoushan farmed population. This gene which play critical roles during the muscle regeneration process^22^. This mutation may be associated with the living environment of the culture cage, where physical friction often occurs due to the high density of fish, resulting in fin injury and regeneration.

We identified nine downstream mutations of ice2 gene, which is involved in cold acclimation and determines freezing tolerance^17^. The gene expression level of ice2 might be affected by these mutations. Since this batch of samples comes from the freeze-resistant selection breeding of Zhoushan domesticated population, variation was found in the gene ice2, which is involved in cold acclimation and determines freezing tolerance.

As the results show, most of the highly enriched GO terms were associated with biological processes and were not shared between the four populations; cellular components and molecular function were also completely different. This result highlights the differences between the croaker populations in different living environments and expands our knowledge of the aquaculture of teleosts.

In this study, we compared the genomes of the individuals from farmed population and those from sea-captured population as described above. There are several points to be aware when interpreting the results of these analyses. First, we collected samples of farmed fish from a population at Zhoushan and another population at Ningde. It is possible that single farmed populations do not well represent the aquaculture populations in both areas. The second point is related to the effect of genetic drift on the allele frequency. The bottleneck effect during domestication process can lead to strong genetic drift, resulting in a drastic change in allele frequencies. In this way, genetic drift can be a potential confounding factor when we try to find putative genes under selective sweep. It should be noted that short generation time and a large number of offspring in each generation can also contribute to the change in allele frequencies. Future studies in which more farmed populations in both areas are included will further elucidate the genomic signatures of adaptation to domestication of large yellow croaker.

Genetic distinctness of Zhoushan farmed population from other three populations should be carefully interpreted. The Zhoushan farmed population has larger linkage disequilibrium blocks than the other three groups (Fig. 3a), and is markedly isolated from the other three populations in PCA and UMAP (Fig. 1b,c). This result indicates the possibility that a part of the Zhoushan farmed fish were not derived from Zhoushan sea-captured population. However, in admixture analysis, some individuals from Zhoushan sea-captured showed highly similar genetic component to those from Zhoushan farmed population (colored in orange in Fig. 2c). It is possible that a small number of individuals have been from the Zhoushan sea-captured population during the process of domestication and subject to selective breeding. In this way, the splice donor mutation of anxa2a may have been selected in Zhoushan farmed.

In conclusion, although the genetic diversities of the four populations is low, plenty of genetic variation for the adaptation to the living environments were obtained. Furthermore, the variation signals of domesticated croaker were found to be stronger than those of croakers in the natural sea environments.

## Methods

### Source of the samples

A total of 198 large yellow croaker were sampled from two provinces (Zhejiang and Fujian Province). The Zhoushan sea-captured individuals were caught by trawler around the Zhongjieshan islands (August–September 2019; latitude 30.198; longitude: 122.682); the Ningde sea-captured individuals were captured around Xiyang Island (November–December 2019; latitude: 26.508; longitude: 120.53; Fig. 1). The Zhoushan farmed individuals were collected from the institute of Administration of Ocean and Fisheries of Zhoushan (August 2019), while the Ningde farmed individuals were collected from Ningde Fufa Aquaculture Co., Ltd. (February 2019). Further details are shown in Table S1. The fish of farmed groups were anesthetized by immersing in MS222 (Tricaine methanesulfonate) prior to tissue sampling as required. All procedures were conducted in accordance with the Regulations for the Administration of Laboratory Animals (Decree No. 2 of the State Science and Technology Commission of the People's Republic of China, November 14, 1988), and approved by the Animal Ethics Committee of Zhejiang Ocean University (Zhoushan, China).

### Genomic DNA extraction

For every sample, 200 mg of tissue was cut into pieces by a knife and transferred to a preheated (56 °C) 2.0 mL tube with 1 mL lysis buffer containing 2 mg proteinase K. The tube was incubated at 56 °C for 1–2 h, then centrifuged at 18,213*g* at room temperature for 10 min. The supernatant was extracted vigorously with one volume of phenol/chloroform/isopentanol (25:24:1) followed by centrifuging at 18,213*g* at room temperature for 10 min in a new tube. The supernatant was transferred into another new tube with 2/3 volume of isopropanol and 100 μL sodium acetate trihydrate followed by gently mixing. The mix was put at − 20 °C for more than two hours and then was centrifuged at 18,213*g* for 20 min at RT and the pellet was washed with 1 mL 75% ethanol. The ethanol was removed by centrifuging and air-drying the pellet for several minutes. The pellet was dissolved in 30–200 μL TE buffer and used for preparation of the sequencing library.

### Library construction and sequencing

After extraction of the genomic DNA, we generate random fragments of the genomic DNA by using ultrasonicator (Covaris). Then, the genomic DNA fragments with an average size of 200–400 bp were obtained with Agencourt AMPure XP-Medium kit. Selected fragments of genomic DNA were end repaired and 3′ adenylated, followed by adaptor ligation to the ends of these 3′ adenylated fragments. The ligation DNA products were amplified by PCR and purified by the Agencourt AMPure XP-Medium kit. The purified PCR products were denatured to single strand DNA by heating, and circularised by the splint oligo sequence. The single strand circle DNA (ssCir DNA) were regarded as the final library and qualified by QC. We sequenced the final qualified libraries by using MGISEQ-2000 sequencer. An ssCir DNA molecule formed a DNA nanoball (DNB), which contains more than 300 copies by rolling-cycle replication. We loaded the DNBs into the patterned nanoarray by using high density DNA nanochip technology. In the end, we obtained pair-end 150 bp reads by using combinatorial Probe-Anchor Synthesis (cPAS).

### Read trimming and mapping

The raw reads obtained were trimmed using Trimmomatic v0.38 (LEADING:30 TRAILING:30 SLIDINGWINDOW:4:25 MINLEN:50)^23^. The reference genome sequence of large yellow croaker was downloaded from Ensembl Release 100 (http://www.ensembl.org), and indexed for BWA v0.7.16a-r1181^24^. The filtered reads were aligned to the large yellow croaker reference genome by BWA-MEM with default parameters.

### Variant calling, filtering and annotations

Variant calling was performed using samtools mpileup with default parameters^25^. To obtain high quality SNPs, only biallelic SNPs were analysed further. Variants with a call rate < 100% and minor allele frequency < 5% were filtered out. To perform variant annotations, the large yellow croaker genome annotation was downloaded from Ensembl Release 100. SNPs were annotated with SnpEff v4.2^26^. Finally, we retained 6,302,244 SNPs as the initial dataset for the downstream analysis.

### PCA and UMAP analysis

The SNP data in VCF format was converted to PLINK binary format using PLINK v1.90b4.5^27^. PCA was then carried out with PLINK. UMAP was performed on the top five principal components with the R umap library.

### Construction of the neighbour-joining tree

To construct a neighbour-joining phylogenetic tree of the samples, we calculated pairwise genome-wide identical-by-state (IBS) distances based on the SNPs using PLINK. Based on the pairwise distance matrix (1-IBS), a neighbour-joining tree was constructed using MEGA7^28^.

### Admixture analysis

The admixture analysis was performed with ADMIXTURE v1.3.0 software^29^. CV errors were estimated for each K-value. The K-value with the lowest CV error was regarded as optimal for estimating the level of admixture in each sample.

### Analysis of effective population sizes

The effective population size was calculated for each group using SNeP v1.1^30^. The parameter for maximum number of SNPs per chromosome was set at 10,000.

### Fst analysis

Fixation index (Fst) values between populations were calculated for all SNPs using PLINK. Fst values more than 0.25 were regarded as significant. Also, average Fst values were calculated using a 40 kb window with 10 kb step. Average Fst values more than 0.05 were regarded as significant. Significant regions were merged and the genes in these regions were reported.

### Gene ontology analysis

Gene ontology (GO) analysis was performed by Metascape^31^ with the following parameters: Min Overlap = 3, Min Enrichment = 1.5, P cut-off value = 0.05. Input gene lists were analysed as zebrafish. The p-values were adjusted for multiple comparisons.

### Ethics approval and consent to participate

The farmed fish were reared in a nucleus farm named ‘Fisheries research institute of Zhoushan’ in Zhoushan City, Zhejiang Province, P.R. China and Fufa Aquaculture Co. Ltd in Ningde City, Fujian Province, P.R. China, respectively; The Zhoushan sea-captured individuals were caught by trawler around the Zhongjieshan islands (August–September 2019; latitude 30.198; longitude: 122.682); the Ningde sea-captured individuals were captured around Xiyang Island (November–December 2019; latitude: 26.508; longitude: 120.53); This study was approved by the Animal Care and Use Committee of Zhejiang Ocean University. All experimental protocols followed ARRIVE guidelines. All participants consented to publish the paper.

### Consent for publication

Consent for publication is not applicable in this study, because there is not any individual person’s data.

## Supplementary Information

Supplementary Table S1.

Supplementary Table S2.

Supplementary Table S3.

Supplementary Table S4.

Supplementary Table S5.

Supplementary Table S6.

Supplementary Table S7.

Supplementary Table S8.

Supplementary Table S9.

Supplementary Table S10.

## Data Availability

The raw reads were deposited in the DDBJ Sequence Read Archive (DRA) under accession number DRP007207. The scripts for the analyses in the current study are available at GitHub (https://github.com/ironman-tetsuo/Whole_genome_resequencing_of_Large_yellow_croaker).

## Abbreviations

Fst: Fixation index
GO: Gene ontology
IBS: Identical-by-state
PCA: Principal component analysis
SNP: Single nucleotide polymorphism
UMAP: Uniform manifold approximation and projection
